# The pandemic agreement: an African perspective

**DOI:** 10.1038/d41586-024-02317-5

**Published:** 2024-07

**Authors:** Nicaise Ndembi, Gerald Mboowa, Sofonias K. Tessema, Yenew Kebede Tebeje, Jean Kaseya

**Affiliations:** Africa Centres for Disease Control and Prevention, Addis Ababa, Ethiopia.

Your Editorial on efforts to agree a global pandemic treaty (*Nature* **629**, 727; 2024) emphasizes that genomic surveillance and pathogen and data sharing are key to effective infection control and development of therapies.

With investment spurred by COVID-19, more than 70% of African Union (AU) member states have developed local capacity to sequence pathogen genomes (M. Makoni *Lancet Microbe* **1**, e318; 2020), and more than 170,000 genomes of the virus SARS-CoV-2 have been sequenced and shared. In collaboration with AU member states, the Africa Centres for Disease Control and Prevention (CDC) is creating a biobanking network across the continent to address pathogen sharing.

Led by the AU and the Africa CDC, a Common African Position on Pandemic Prevention, Preparedness, and Response was adopted in February 2024 (see go.nature.com/3xqvrhs). Agreement on a pathogen-access and benefit-sharing system — which will allow rapid exchange of sequence data and equitable and timely access to countermeasures for pandemic preparedness and response — will be the most crucial outcome of the pandemic treaty. Sharing of pathogen data, reciprocated with multilateral benefits, can provide a guarantee of safety and equity for people all over the world, whether in higher- or lower-income countries.

